# Numerical Simulation and Optimization of Furnace Roll Casting Production Technology

**DOI:** 10.3390/ma18235445

**Published:** 2025-12-03

**Authors:** Martina Bašistová, Filip Radkovský, Petr Lichý, Šimon Kielar, Iveta Vasková

**Affiliations:** 1 Department of Metallurgical Technologies, Faculty of Materials Science and Technology, VSB-Technical University of Ostrava, 17. Listopadu 2172/15, 708 00 Ostrava, Czech Republic; filip.radkovsky@vsb.cz (F.R.); petr.lichy@vsb.cz (P.L.); simon.kielar@vsb.cz (Š.K.); 2 Department of Metallurgy, Faculty of Materials Metallurgy and Recycling, Technical University of Kosice, Letná 1/9, 042 00 Košice, Slovakia; iveta.vaskova@tuke.sk

**Keywords:** casting technology, pouring simulation, solidification, cast steel, nodular cast iron, porosity, defects prediction

## Abstract

This study investigates the use of steel and cast iron for producing cast furnace rolls to replace welded rolls, which often fail from cracks and limited durability. Casting had not been previously considered by the manufacturer, but rising demands for durability and quality make it a promising alternative. Material selection focused on mechanical properties, wear resistance, and production costs. To ensure casting quality, Magmasoft 6.0 software was applied for detailed simulation of casting, solidification, and cooling. Results showed that steel alloys (GS-34CrMo4 and GS-20Mn5) are prone to shrinkage and porosity, which cannot be fully avoided even with feeders. In contrast, GJS-500-7 cast iron exhibited low shrinkage tendency and minimal defects, proving suitable for production while reducing costs. It also offers lower weight and efficient metal use, improving cost-effectiveness. Detected defects were concentrated in the central casting area, where they have little impact on functionality. Based on sixteen simulations, GJS-500-7 cast iron emerged as the most suitable material for furnace rolls thanks to its thermal resistance, castability, low porosity, and ability to meet required specifications. This process optimization represents an efficient, cost-effective choice, improving final product quality and creating new opportunities for the manufacturer.

## 1. Introduction

Furnace rolls operating in zones with low thermal gradients and minimal material residence time in the furnace (entry/exit sections of continuous annealing lines, tempering zones at ~580–660 °C, transition to the run-out table) function under short-term thermal exposure and lower mean temperature. Zonal temperature control in CAL/CGL systems (heating/even-heat/cooling) reduces the accumulation of creep damage and thermal-fatigue cycles in the rolls, allowing the use of simpler material solutions compared to high-temperature soaking sections [1]. In these zones, welded roll constructions (shell + ends + shafts) are widely applied; their failures are typically governed by local thermal expansion, repeated contact shocks, and residual stresses in weld regions. Numerical and operational analyses identify the dominant mechanism as creep-fatigue with stress concentration in the weld root and toe [2,3].

For load-bearing welded rolls in “cold-end” zones, quenched and tempered low-alloy steels such as 34CrMo4 (or 35CrMo) are commonly used, providing a favorable combination of strength and toughness and good impact response; however, with increasing temperature, temper softening and carbide precipitation occur, limiting long-term strength above ~650–700 °C [4,5]. Cast steel GS-20Mn5 is used for auxiliary or idler rolls and less-loaded assemblies; its viscoplastic response and grain evolution at elevated temperature make it suitable for shorter exposures rather than long-term soaking [6]. Ductile iron GJS-500-7 offers good impact damping and castability, but between 400 and 700 °C, its strength and low-cycle fatigue (LCF) life decline rapidly due to oxidation and stress concentration at graphite nodules. Its use is therefore reasonable only in the coldest, short-exposure areas, or in SiMo variants that remain stable up to ~700–750 °C [7,8].

Mechanical loading in these zones is dominated by rolling contact with short thermal impulses. Key material parameters include temper-softening resistance (for 34CrMo4), toughness, and weld joint resistance to combined creep-fatigue. Experimental and FE studies on welded components show that differences in properties between weld metal and heat-affected zones increase local creep deformation and accelerate crack initiation; proper filler selection, controlled thermal cycles, and post-weld heat treatment (PWHT) reduce residual stresses and extend service life [3,9]. From the tribochemical standpoint, surface wear is determined by the formation of early oxide layers (FeO/Fe_3_O_4_) and the transfer of oxides from the strip; short exposures limit measurable scale growth, though repeated thermal shocks generate stresses in the oxide and micro-spallation, which increase roughness and friction [10]. To limit pickup and stabilize the surface, thin HVOF or VPS coatings based on NiCoCrAlY (optionally alloyed with Hf or Si) are successfully used in practice; within 600–900 °C, they suppress oxidation and improve cyclic adhesion [11].

Typical defects of welded furnace rolls in “cold-end” zones include the following: (i) initiation and propagation of cracks near welds due to combined creep-fatigue, (ii) local bulging and bending under prolonged exposure above design temperature, (iii) micro-spallation of surface scales leading to increased friction, and (iv) for cast irons—subcritical cracks initiated at graphite nodules and a temperature-dependent decrease in fracture toughness [2,3,7]. Case studies of related furnace components (supports, tubes) confirm the same failure mechanisms: thermal non-uniformity, material ageing, and intergranular weakening leading to premature failure [12]. Integrating structural optimization (load redistribution among rolls, cooled journals, shorter spans) with appropriate material and welding technology demonstrably extends maintenance intervals in entry roll assemblies [2].

Rolls cast with internal shrinkage cavities or porosity exhibit reduced service life because voids act as preferential crack initiation sites, lowering the fatigue limit and accelerating crack propagation. The risk increases with defect size and proximity to the surface, while the scatter in lifetime rises markedly [13]. In cast steels, the direct influence of central and off-axis shrinkage on the decrease in low-cycle fatigue life has been experimentally demonstrated—larger cavities lead to faster crack initiation and shorter life [14]. A similar trend applies to cast irons: the presence of shrinkage defects significantly reduces fatigue strength, with fissured shrinkage pores being more detrimental than gas porosity [15]. At elevated temperatures, where creep interacts with fatigue, such defects promote earlier cavity formation and intergranular weakening, accelerating failure of cast rolls in furnace service [16].

Critically, in low-temperature and short-exposure sections, combining welded steels 34CrMo4 (for load-bearing and drive rolls) and 20Mn5 (for idler rolls) provides sufficient operational reliability and cost efficiency, provided that proper heat treatment (Q&T), cooling, and atmospheric control are maintained [4,6]. For 34CrMo4, literature confirms a stable strength–toughness balance at tempering temperatures around 580–650 °C, while long exposure above 700 °C promotes carbide precipitation and hardness loss [4,5]. Conversely, cast steel 20Mn5 shows good plasticity and resistance to short thermal impulses, making it suitable for less-loaded rolls in colder furnace zones [6]. Ductile iron GJS-500-7 can be used selectively where impact damping and short material contact are critical; at 400–700 °C, its strength and fatigue life decrease sharply due to oxidation and stress at graphite nodules [7]. Transitioning to modified SiMo grades shifts usability limits toward ~700 °C, with improved oxidation resistance and longer surface life under cyclic heating [8]. Operational strategy remains conservative: heat-resistant austenitic alloys are reserved for hot furnace sections [2,12], while welded rolls from standard low-alloy steels are efficiently employed in cold-end zones with verified fatigue and surface stability, where mechanical and thermal loads remain limited [1,9,11].

Numerical simulations are increasingly used to enhance quality and improve efficiency in foundry production. Combined with experiments, simulations provide greater flexibility, cost savings, and help to prevent the origin of casting defects or optimize production technology [17,18]. One of the most widely used programs in European foundries is Magmasoft, which monitors processes such as mold filling, solidification, and casting cooling. It also enables internal stress analysis, helping to prevent deformation [19]. For solidification simulations of castings in metal molds, the finite element method (FEM), finite difference method (FDM), and boundary element method (BEM) can also be used [20]. Numerous other foundry defects can be predicted through precise modeling of temperature profiles, allowing optimization of technological processes to prevent or mitigate defects [21,22,23]. Accurate thermophysical data for various materials play a crucial role, as simulation results are highly dependent on the quality of material data. It is therefore also possible to use programs or databases such as Thermo-Calc^®^ and JMatPro^®^ [19,24] or the SimulationDB database [25].

Casting process simulations reveal major findings on casted alloys. Studies [26,27] analyzed ductile iron (GJS-400) castings, simulating metal inflow, solidification, and cooling with chills, with experimentally determined thermophysical properties enabling microstructure and stress evaluation. Works [28,29] examined gray iron microstructure, grain morphology, and gating design, integrating simulation with production, economic, and environmental aspects. Research [30,31,32] focused on optimizing ductile and Si-Mo ductile iron casting quality through combined simulation and experimental validation, addressing heat transfer, cooling rate effects, and residual stress prediction for casted parts working in high-temperature applications above 750 °C. Studies on steel casting simulations addressed heat release [33], thermal databases [34], mold cooling, predicting temperature fields, reducing internal stresses [35,36], and chiller design, improving accuracy, yield, and solidification efficiency [37], or optimizing mold materials and bimetal casting process [38]. Reduced cooling performance during thermal cycling clarified irreversible sand behavior in ductile iron casting. With increasing demand for sand casting, modern control methods such as 3D printing and wireless sensors enhance data acquisition and model validation [39]. Research [40,41] focused on optimizing mold lifespan and minimizing microporosity through combined experimental and simulated analysis.

The novelty of the numerical modeling approach lies in the targeted use of simulations to determine the most suitable production technology and material for an already industrially applied furnace roll with a fixed geometry. The modeling process was designed not only to predict typical casting defects such as shrinkage and porosity but also to optimize the gating and feeding system within existing design constraints, where major shape modifications were not permitted. This approach allowed for a direct comparison of different materials and technological variants, linking numerical results with real manufacturing feasibility. The aim was to achieve a balance between casting quality, technological efficiency, and cost-effectiveness, demonstrating a practical and innovative use of numerical modeling in optimizing complex foundry components under real industrial conditions.

This study focuses on the use of steel or cast iron for producing castings that will serve as furnace rolls, intended to replace the currently manufactured welded rolls. The current version, produced by welding, is highly susceptible to defects, most commonly cracking during use. Therefore, transitioning to castings presents a promising alternative solution that has not been considered by the current manufacturer. In selecting a suitable material, various factors needed to be considered, such as mechanical properties, wear resistance, and production costs. To achieve the most efficient and high-quality manufacturing process, simulation software Magmasoft 6.0 will be utilized. This software enables detailed analysis and simulation of casting, solidification, and cooling, which may help optimize process parameters and select the most suitable material for producing furnace rolls in the future. By using numerical modeling, it is possible to predict and minimize potential defects in actual castings, potentially leading to improved overall product quality and reduced production costs. Optimization using this advanced simulation tool is essential to ensure that selected materials and manufacturing processes meet the required standards and specifications for producing furnace rolls. The novelty of this research lies primarily in proposing a new solution for a specific supplier who has not previously considered this production option.

## 2. Materials and Methods

### 2.1. Design and Modification of the Furnace Roll Structure

**Figure 1:**
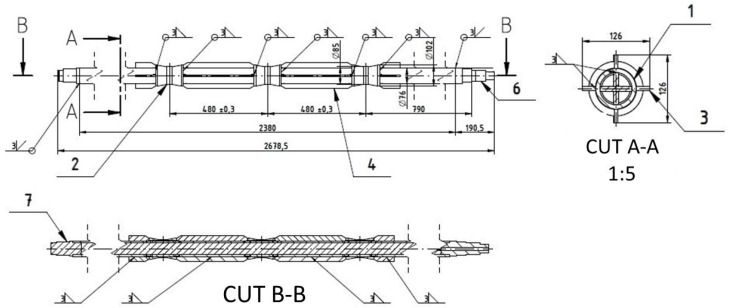
Current technical drawing (sketch) of furnace roll shape.

The original rolls were manufactured as welded constructions from several dissimilar parts, some of which were thin-walled and made from various steel grades. During use, these rolls were subject to cyclic heating and cooling, and due to uneven wall heating or weld quality, they often developed irreparable defects. As a potentially more cost-effective alternative, a design for the production process using foundry methods was chosen, with the suitability of this production approach first requiring validation through simulations. Initially, attention was given to the design of the roll itself. Both the existing technical drawings (Figure 1) and the furnace manufacturer’s designs were considered.

**Figure 2:**
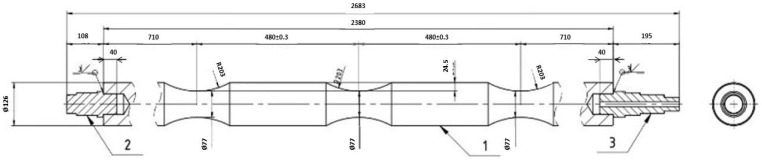
Revised drawing for the casting of a furnace roll shape.

Both were thoroughly reviewed, and a new design was created that accounted for the conditions of use and additional user requirements for the rolls’ intended application, without allowing significant modifications to the entire structure. The design was only slightly adjusted for the casting technology, with an emphasis on eliminating sharp transitions that could lead to stress concentration and create hotspots. Such areas could pose issues during production and use. The design was further optimized to best fit the foundry casting technology and facilitate production (Figure 2). The restrictions on design changes allowed only for choosing between one or two runners and adjusting the shape and volume of feeders. Emphasis was placed on ensuring that, upon completion, the roll would not be susceptible to defects caused by stress or other factors. The goal was to create a simple shape without sharp transitions that could potentially cause issues in the future.

### 2.2. Designing of Casting Position in the Mold and 3D Modeling

When designing the placement of the casting in the mold, three options were considered. The first option was the right centrifugal casting technology, which was ultimately dismissed as unfeasible. The main reason was the small diameter of the furnace roll (126 mm) combined with its considerable length (2380 mm). With this technology, issues with insufficient metal feeding in the central section of the roll could arise, which might typically be resolved with feeders. However, with such a small diameter, feeders would be less effective, resulting in a high risk of cavities and other defects.

The second option was stationary vertical casting, typically with bottom bottom-gating system entry, placing the roll in a vertical position. The advantage of this method is the smooth filling of the mold cavity and good chemical homogeneity of the material across its cross-section. However, given the length of the roll, this technology posed challenges due to the need to assemble the mold from multiple parts, complicating accurate geometry maintenance and subsequent defect correction.

The third and selected option was classical horizontal gravity casting into a split sand mold with lateral entry of the gating system. Metal transport into the mold cavity was ensured by runners along the sides of the roll, combined with a series of gates to facilitate smooth and sufficiently rapid filling.

**Figure 3:**
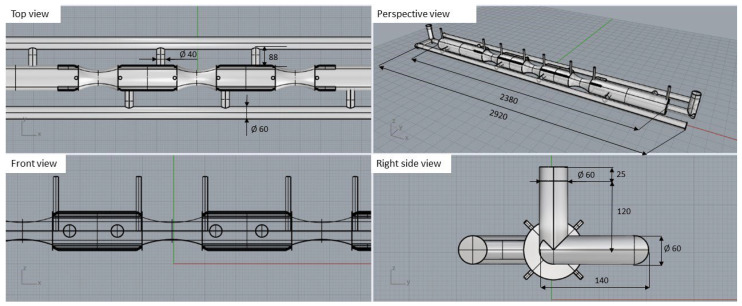
Design of roll casting and created 3D models in Rhinoceros 6.0 software.

**Figure 4:**
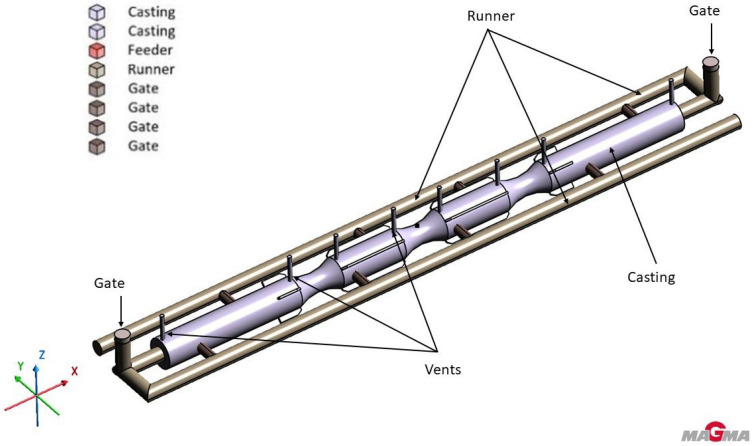
Geometry of the casting and one variant of gating system.

The 3D modeling of the furnace roll, along with the proposed gating system and feeder options, was carried out using the 3D modeling software Rhinoceros 6.0. This surface-based 3D NURBS modeling program is commonly used in technical fields and design. The various solution options and completed 3D models are shown in Figure 3, and the geometry with the gating system, runner and vents is presented in Figure 4.

**Figure 5:**
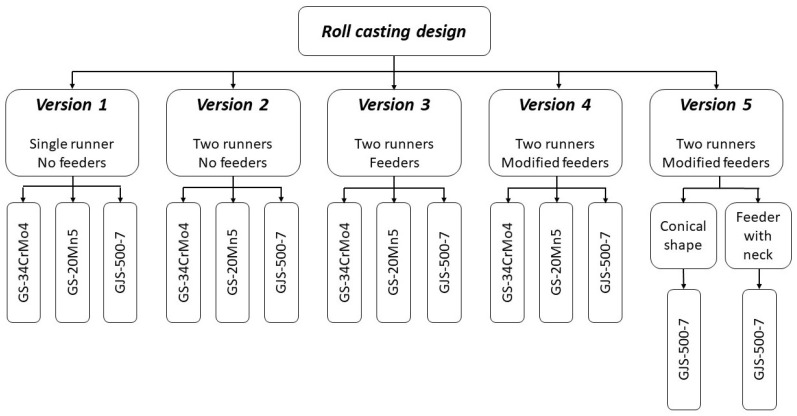
Schematic representation of the simulations performed.

In designing the gating system, attention had to be given to potential problems arising from the casting’s structure. The primary concerns included inadequate filling speed of the mold cavity, which could lead to misrun defects or freezing of the flow front, or conversely, gas entrapment, potentially causing similar issues or bubble formation in the casting. Neither of these defects was acceptable. Two variations were considered in the gating system design: one using a single runner with five separate gates, and the other with two runners along both sides of the casting and ten gates, with each runner supplying metal from each side of the roll, filling simultaneously from both ends. The size, shape, and volume of the feeders were evaluated based on specific simulation results and adjusted as needed for subsequent simulations. A schematic representation of the simulations performed is visible in Figure 5.

### 2.3. Selection of Casting Material for Furnace Roll Simulations

Based on the required properties—primarily reducing the risk of cracks or fractures in the casting due to cyclic thermal stress—three casting alloys were selected for the simulations. Each material was chosen for its mechanical properties and potential advantages. The materials included cast steels GS-34CrMo4 (according to DIN 17205:1992 [42]), to replace the usually used welded steel 34CrMo4, and cast GS-20Mn5 (according to DIN EN 10293:2015 [43]), as well as ductile iron GJS-500-7 (according to EN 1563:2018 [44]). The steels for castings were chosen primarily based on their superior mechanical properties and durability compared to cast iron. The specific reasons for selecting each material, as well as any potential disadvantages, are provided below.

The alloyed steel GS-34CrMo4, compared to GS-20Mn5, offered primarily higher tensile strength, with values per standards ranging from 620 to 800 MPa. A roll made from this material would better withstand loading and stress, potentially extending its service life. The material’s hardness reached 255 HB. However, this material would not be ideal for hardening, as it might be prone to notch formation and cracking. Its disadvantages also included poorer weldability.

For the manganese steel GS-20Mn5, the primary reason for selection was its excellent weldability, which would potentially allow for quick and easy repairs of the casting in case of damage during use. Its mechanical properties included a tensile strength ranging from 500 to 650 MPa, an impact toughness of 50 J/cm^2^, and a hardness of 140–180 HB.

Ductile iron GJS-500-7, compared to the previous materials, exhibited an average tensile strength of 420–500 MPa according to standards. Its main advantages were generally excellent castability and lower susceptibility to volumetric changes compared to steel. These properties would be beneficial in the roll’s manufacturing process, whether during casting or subsequent machining. Additionally, ductile iron featured low specific weight and could often forgo heat treatment.

**Figure 6:**
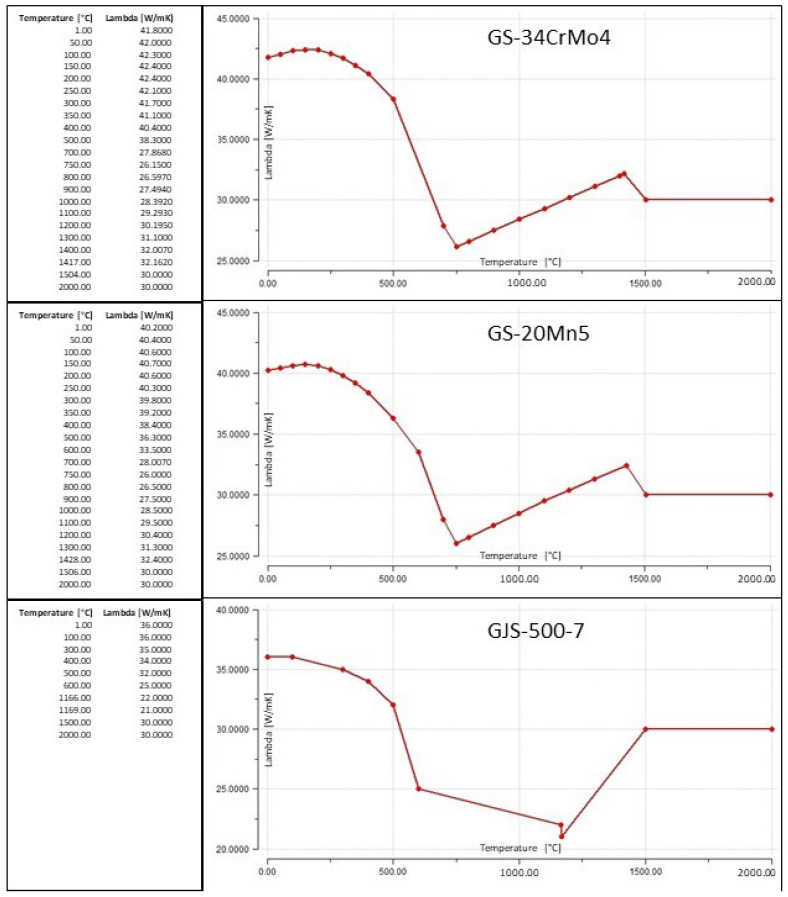
Lambda as function of temperature from simulation database.

The thermophysical properties of materials are shown in Table 1. Conductivity, viscosity, and other properties depend on temperature. This dependence Lambda (λ), used directly in simulations, is shown in Figure 6.

### 2.4. Simulation Parameters

Each proposed version is based on the partial results of the previous simulated version. The goal was to achieve improved casting quality and increased utilization of liquid metal in the next simulation version. Specifically, the aim was to reduce internal porosity, relocate it to a less critical area near the geometric axis of the furnace roll, or ideally, eliminate it entirely.

With each successive series, the primary changes and modifications focused on adjusting the gating system and modifying the feeders. The resulting parameters of each simulation version, as they progressed chronologically, along with the degree of changes in each solution, are shown in the overview Table 2. Notably, there were changes in the feeders and pouring time. The weight of the castings themselves, excluding gating systems and feeders, remained essentially constant, with only minor differences due to internal porosity.

The FVM method was always used for network design and evaluation. The Finite Volume Method (FVM) divides the entire geometry into individual sections—referred to as cells—for the purpose of numerical calculation. In Magmasoft, these cells typically take the shape of cubes or rectangular prisms. The mesh cell size used in the casting region was a cube with dimensions 5 × 5 × 5 mm, while in the gating system, the maximum cell size was 5 × 5 × 10 mm. Across the entire model, the prevailing cell size remained at 5 mm, maintaining consistency in mesh resolution. Refining the mesh contributes to a more accurate and reliable simulation, as it enables better detection of gradients and local variations between adjacent cells. This increased level of detail allows the simulation to capture subtle physical phenomena more precisely. However, the improvement in accuracy comes at the cost of increased computational time. The total number of metal cells within the simulation ranged from approximately 260,000 to 370,000, depending on the specific design of the casting and gating system. The quality of the mesh is assessed graphically, focusing on parameters such as Thin Walls, Blocked Cells, and Edge-to-Edge connections. In the evaluated meshes, only a few isolated problematic cells were identified. Given their low number and non-critical location, these cells had no significant impact on the overall accuracy of the simulation. Specifically, the number of problematic cells ranged from 26 to 54 for thin walls, 0 for blocked cells, and between 0 and 12 for edge-to-edge connections. Based on these values, the mesh was consistently evaluated as high-quality. In this case, the evaluation was conducted for the entire geometry, with special attention given to the thinnest regions of the casting—particularly notches and blade sections—where finer resolution is essential to accurately capture complex flow behavior and thermal gradients.

### 2.5. Mathematical Formulations

The mathematical data (formulas, algorithms) are embedded in the software and are not publicly available. General principles are known, but how they are implemented or adapted is not disclosed. The user can only influence input data, such as initial temperatures, material type, heat transfer, etc. To discuss the general principles and methods used by Magmasoft, it is possible to examine the finite volume method (FVM). Below are the formulas that need to be established before using simulation software Magmasoft 6.0 and that are essential for performing preliminary calculations. Based on these results, the basic dimensions of the gating system, feeders, etc., are designed. The basic Equations (1)–(5) used are as follows [45,46]:

Calculation of pouring time according to Dietert:

(1) $$\tau =s\times \sqrt[{3}]{m_{h}}\;\;\;[s]$$

where *s* is the coefficient chosen based on the wall thickness of the casting [mm] and *m_h_* je is the raw weight of the casting [kg].

Calculation of the average metallostatic pressure if the notches are placed at the center of the casting:

(2) $$h_{mid}=H-\frac{P^{2}}{2c}\;\;\;[m]$$

where *H* is the height between the molten metal level in the pouring basin and the center of the notches [m], *p* is the height of the mold cavity above the notches [m], and *c* is the height of the total mold cavity [m].

Calculation of the controlling cross-section area:

The controlling cross-section is most often the notch, which is the smallest cross-section in the gating system.

*S_z_*:*S_r_*:*S_k_*/1:1.1:1.2

where *S_z_* is the area of the notch [m^2^], *S_r_* is the area of the distributing channel [m^2^], and *S_k_* is the area of the lower dimension of the gating sprue [m^2^].

The area of the notch is then calculated using the Equation (3).

(3) $$S_{z}=\frac{m}{\tau \times \mu \times \rho \times \sqrt{2gh_{mid}}}\;\;[m^{2}]$$

where *m* is the mass of the casting with additions [kg], *τ* is the total pouring time [s], *µ* is the resistance coefficient against flow (0.38 for non-ferrous metals), *ρ* is the density of the poured metal [kg/m^3^], *g* is the gravitational acceleration [m/s^2^], and *h_mid_* is the average metallostatic pressure [m].

Calculation of the feeder volume:

The feeder volume is a critical parameter for properly adding metal, and its value is calculated using Equation (4). Often, this value is only an approximation.

(4) $$V_{n}=\frac{x\times \beta }{1-x\times \beta }\times V_{o}\;\;\;[m^{3}]$$

where *x* is the feeder inefficiency coefficient, *β* is the coefficient of volume shrinkage of the alloy, and *V_o_* is the volume of the casting [m^3^].

Calculation of liquid metal utilization:

(5) $$ɩ\;=\frac{m_{o}}{m_{s}}\times 100\;\;\;[\%]$$

where *m_o_* is the gross weight of the casting–with allowances for machining [kg] and *m_s_* is the raw weight of the casting, including the gating system, feeders, and vents [kg].

## 3. Results and Discussion

### 3.1. Version 1: Gating System with Single Runner

**Figure 7:**
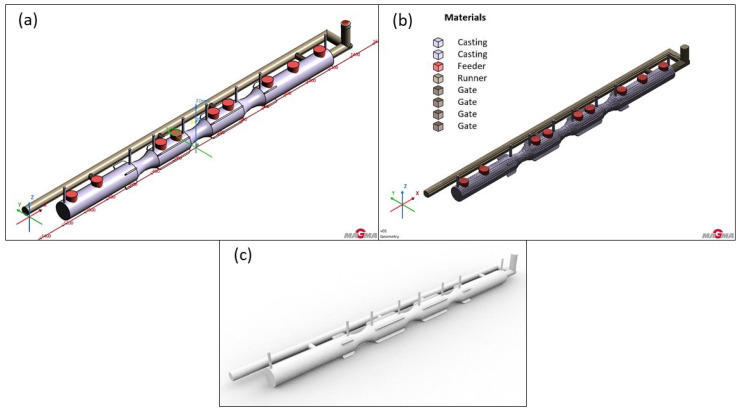
Version 1: Single runner design: (**a**) geometry; (**b**) mesh; (**c**) shape of the casting with the gating system and vents.

In version 1, a single runner resembling the letter “L” was used, along with five gates (see Figure 7). The subsequent entry was achieved through direct and lateral connections to the future casting. The length of the runner matched the length of the furnace roll. This setup aimed for even filling of the mold cavity, along with sufficient speed and better utilization of liquid metal compared to the two-runner version.

**Figure 8:**
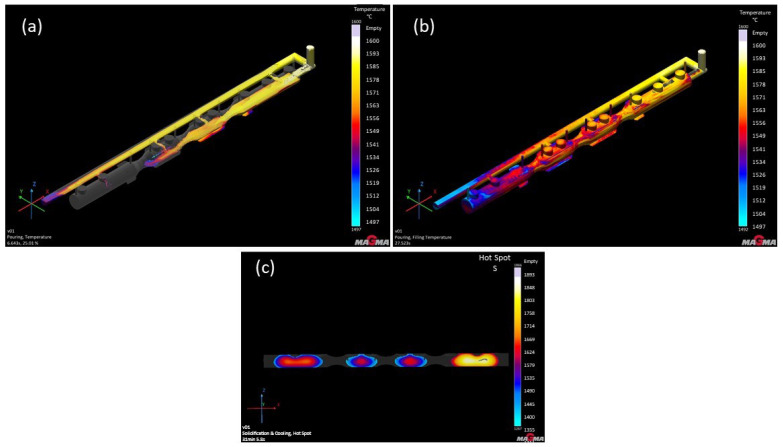
GS-34CrMo4 version 1: (**a**) pouring; (**b**) solidification and cooling; (**c**) hotspot result.

**Figure 9:**
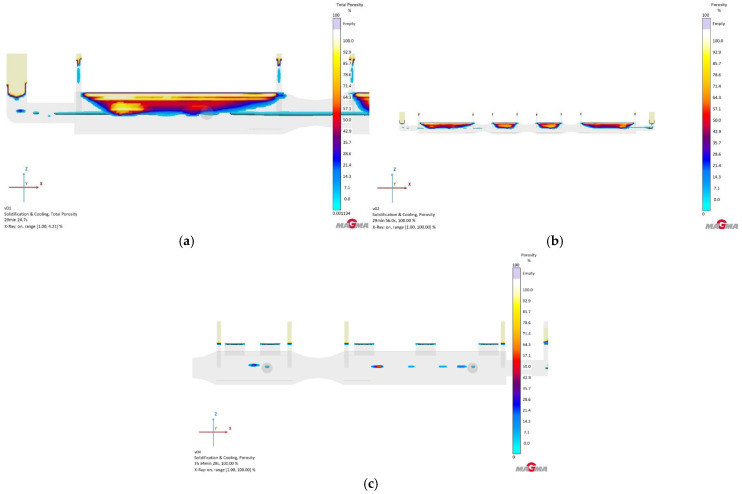
Total porosity results of version 1: (**a**) GS-34CrMo4; (**b**) GS-20Mn5; (**c**) GJS-500-7.

In the case of the GS-34CrMo4 material, the metal flow within the mold cavity was somewhat turbulent, which in real conditions could lead to erosion in certain mold areas (Figure 8). Notably, the filling time was relatively long, reaching 11 s. This material was highly susceptible to shrinkage and porosity. Although this susceptibility was anticipated, the level of defects in the casting was disproportionately high. Among the three simulated materials with this type of gating system, the chrome–molybdenum steel likely performed the worst. Figure 9b clearly shows porosity extending almost halfway into the casting cross-section. Higher defect content was represented by various color shades: yellow and beige indicate porosity around 100–70%, red to orange covers the range of 70–42%, while dark blue to blue-green represents 42–0%. This highlighted the need to focus on defect mitigation in future simulations, given the high percentage of these defects.

The simulation result for the single-runner configuration with GS-20Mn5 was comparable to GS-34CrMo4. Although some minor improvements were observable, the focus remained on porosity percentages at various casting depths. It was immediately apparent that the material’s susceptibility to shrinkage was slightly lower than that of Version 1. However, it still posed a significant issue, making it unsuitable for use, as was the case with the first material (Figure 9a).

When simulating the casting and solidification process and defect prediction for cast iron, it is important to note that, concerning volumetric changes and shrinkage, cast iron behaves entirely differently compared to cast steels, a difference confirmed in this experiment. Initially, two grades of ductile iron—GJS-500-7 and GJS-700-3—were considered for simulation. Ultimately, GJS-500-7 was chosen primarily for its lower susceptibility to volumetric changes and shrinkage. The simulation results indicated that the percentage of porosity was minimal, almost approaching zero. Although some minor porosity was observed in the casting’s hotspots, it was negligible compared to the previous two steel simulations (Figure 9c).

The anticipated complications were partially confirmed. Metal flow into the mold was highly turbulent in all cases, with the highest pressure and metal velocity recorded at the first front gate and the second side gate. In these areas, mold erosion could occur, potentially altering the shape of the final casting. Another issue was metal cooling. Given the length and narrow profile of the casting, the single-runner simulation showed a temperature loss of several degrees Celsius as the metal flowed toward the mold’s edge. This cooling was caused by the initial contact of the metal front with the surrounding atmosphere and mold, which further transferred heat, thus cooling it more significantly, potentially leading to issues with metal homogeneity.

Based on the results, several solutions or mitigation strategies were considered. The first option was to pour the metal from a greater height, which would increase the flow velocity and reduce the time the metal front was exposed to heat transfer. However, in real production, this would risk mold erosion and metal splashing, potentially resulting in inhomogeneity or the formation of so-called cold shuts. Another option was to increase the pouring temperature. Although the metal would still cool during flow, it would not reach a temperature that would compromise casting quality. However, a higher pouring temperature increased the risk of shrinkage formation, as the metal would cool more slowly, leading to greater volumetric changes. The final option was to modify the gating system. While this would reduce the utilization of liquid metal, the adjustment could ensure more even filling of the mold cavity with minimal metal cooling. Summary results of this simulated Version 1 are presented in Table 3.

### 3.2. Version 2: Gating System with Two Runners

**Figure 10:**
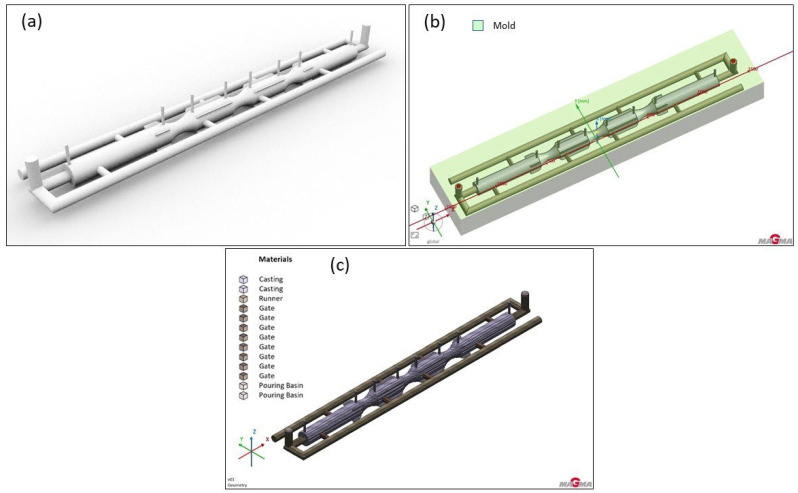
Version 2: Two runners design: (**a**) shape of the casting with the gating system and vents; (**b**) geometry in the simulated sand mold, (**c**) mesh with the gating system.

In the first series of simulations with a single runner, it was observed that the mold cavity filling was not entirely smooth. Although the cavity was fully filled, the pouring time was relatively long. This could pose a problem when using feeders, as they would increase the required metal volume for casting, thus raising the risk of misruns in the boundary areas of the casting or within the feeders. For this reason, a variant with two runners was also tested (see Figure 10). Although the utilization of liquid metal was 7% lower, the cavity filling was more uniform and faster. In the previous version, it was mentioned that porosity would need to be addressed with feeders; however, feeders were intentionally omitted in this version. The aim was to verify whether the gates alone could partially act as feeders, helping to compensate for volumetric changes during solidification. The goal was to determine if doubling the number of gates would impact the casting’s porosity outcomes.

**Figure 11:**
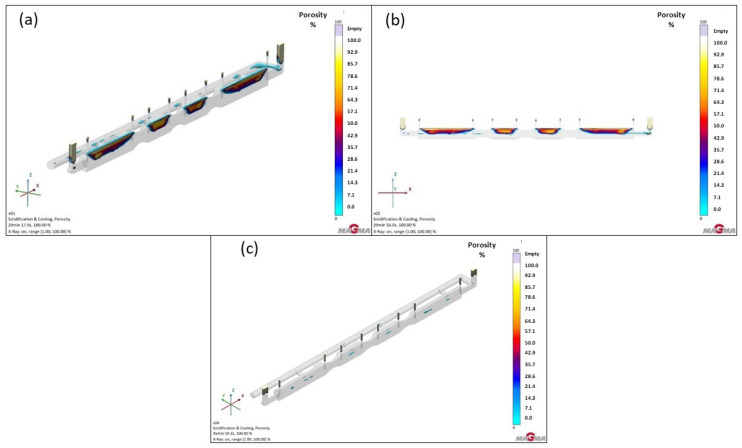
Total porosity results of version 2: (**a**) GS-34CrMo4; (**b**) GS-20Mn5; (**c**) GJS-500-7.

Although the change in the gating system was expected to reduce porosity in the casting made from GS-34CrMo4, there were no significant improvements, as seen in Figure 8a. Porosity was slightly reduced, but no substantial shift occurred. Using such a roll in real operation would lead to a very short service life, likely ending with the formation of cracks and subsequent fracture. Similarly, there were no significant changes in the GS-20Mn5 variant (see Figure 11b). The porosity in the central part of the casting decreased only by a few percentage points. This roll would be unsuitable for use. Future simulations with GS-20Mn5 and GS-34CrMo4 will need to incorporate feeders, as both materials exhibit a high tendency toward shrinkage, making the use of feeders essential. Although the previous GJS-500-7 simulation showed minimal porosity in the roll, it was necessary to determine the impact of two gating channels with double the number of gates on the material’s cooling and formation. The gates could partially function as feeders, thereby reducing shrinkage formation. The result was somewhat better than Version 1 (one runner), but still not a major improvement, as seen in Figure 11c. Although porosity was recorded during the simulation, it would most likely not have a significant impact on the roll’s quality and service life.

Unlike the previous series of simulations, two runners with double the number of gates were now used. This increase aimed primarily to allow faster and more uniform metal flow within the mold cavity, as previous simulations suggested that mold erosion might occur, potentially altering the casting’s shape. It was also expected that defects in the form of porosity and shrinkage might at least partially decrease, as the gates themselves could somewhat serve as feeders, supplying metal to critical areas of the casting.

After implementing these adjustments, it was observed that the metal filled the mold cavity significantly better, potentially preventing metal impacts on the mold and subsequent erosion. This improvement was also supported by gating from both sides, with metal flows meeting and merging in the center of the casting. This filling method also reduced the pronounced cooling of the metal front, which was an issue in previous simulations.

However, the expected significant reduction in defects did not occur. Although the gates partially acted as feeders, the feeding was insufficient to fully replace feeders or lead to a notable improvement in quality. Therefore, it was decided that some form of feeding would need to be incorporated into future simulations.

Among the three materials used, both cast steels yielded the poorest results. While the GS-20Mn5 material showed a partial improvement in the percentage of porosity, the changes were not significant. Ductile iron GJS-500-7 performed the best, with porosity observed at only a few percent, which would likely not impact the operational use of the roll. According to standards, this material also exhibited high wear resistance and lower weight or density compared to both grades of steel. Summary results for simulated Version 2 are shown in Table 4.

### 3.3. Version 3: Gating System with Two Runners and Feeders

**Figure 12:**
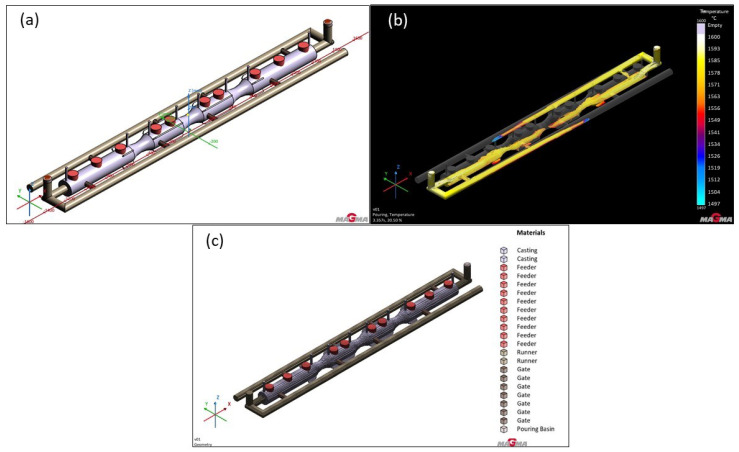
Version 3: Two runners and feeders design: (**a**) shape of the casting with geometry; (**b**) pouring; (**c**) mesh with gating system and feeders.

**Figure 13:**
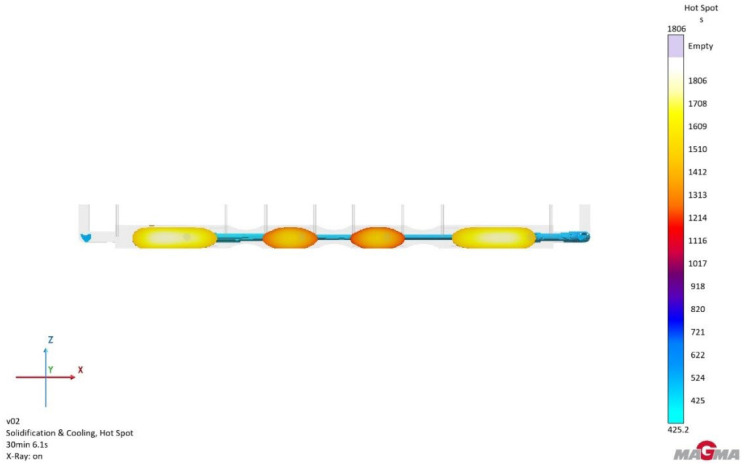
Version 3: areas of hotspots.

Due to recurring issues with excessive porosity in both versions 1 and 2, it was decided to use feeders (see Figure 12). Their purpose was to supply liquid metal to hotspots during the casting’s solidification, specifically targeting areas where the casting solidified last. This would ensure that, as volumetric changes occurred—which are typical for any material—shrinkage would not lead to cavity formation or imperfect material bonding, thus preventing porosity. Hotspots were identified based on simulations (see Figure 13), and feeders were then strategically positioned accordingly.

**Figure 14:**
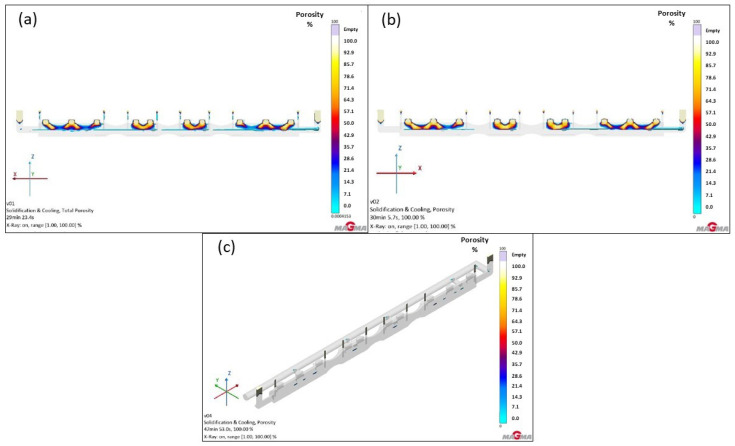
Total porosity results of version 3: (**a**) GS-34CrMo4; (**b**) GS-20Mn5; (**c**) GJS-500-7.

The main change from simulations in Versions 1 and 2 for the GS-34CrMo4 material was the addition of ten individual feeders to the casting, positioned based on hotspot locations. The largest hotspot was located on both end sections of the furnace roll, where the material was thickest, leading to limited heat dissipation. For this reason, feeders were placed in these areas to cover the critical zones and supply an adequate amount of metal. However, after running the simulation, it became apparent that, although the feeders functioned as intended and their volume was effectively utilized to replenish metal in the hotspots, they were significantly undersized and thus insufficient to fully compensate for the shrinkage in the feeder. Their modification will be necessary in the next series of simulations. It should also be noted that this simulated material exhibited the highest susceptibility to volumetric changes among all three materials, as shown in Figure 14a.

Similar to previous simulations with GS-20Mn5, there was no substantial difference from the prior test. However, a notable shift of defects into the feeder was observed, accounting for approximately 30% of the total porosity. Figure 14b displays the percentage distribution: beige areas in the feeders indicate 100% shrinkage, followed by 80–90% marked in yellow. A gradual transition into the casting body is clearly visible, with colors shifting from orange to red and eventually to blue. Orange represents porosity of 70–60%, while red indicates 50%, primarily located in the central casting area. Blue areas correspond to a range of 30–0%, found in and beyond the central casting region. This confirms that the feeders were functional in partially feeding metal into the hotspot, but they were not large enough. Their shape, which did not fully cover the hotspot area, might also have been a factor. Further focus on optimizing this design will be necessary.

As the GJS-500-7 material exhibited very low shrinkage tendency, the issue of volumetric changes was less significant compared to the steels. However, compared to the previous simulation without feeders, a few percentage points decrease in porosity was evident in this case (see Figure 14c). A closer inspection revealed porosity only in the casting center, with the highest percentage reaching approximately 48%. This presence was negligible, as the actual shrinkage dimensions in the casting would likely be around 5 mm in height and approximately 25 mm in length.

Although a completely flawless casting without internal defects has yet to be achieved, the results represent significant progress compared to the initial simulations. Particularly valuable was the improvement in the gating system for such a complex casting shape, allowing for smooth and uniform filling without unwanted anomalies. Thorough monitoring of the simulated material’s behavior now provides a much clearer understanding of its properties, enabling more precise optimization of casting conditions.

Future simulations will therefore focus on modified feeders with a larger volume and a shape better suited to the hotspots to more effectively fulfill their function. An interesting adjustment will also be the modification of runners, mainly to eliminate backflow. This change is intended to prevent the molten metal from hitting the back wall of the channel, as observed in the simulation, which slows down the flow and reduces the efficiency of filling the gates. Summary results of simulated Version 3 are shown in Table 5.

### 3.4. Version 4: Gating System with Two Runners and Modified Feeders

**Figure 15:**
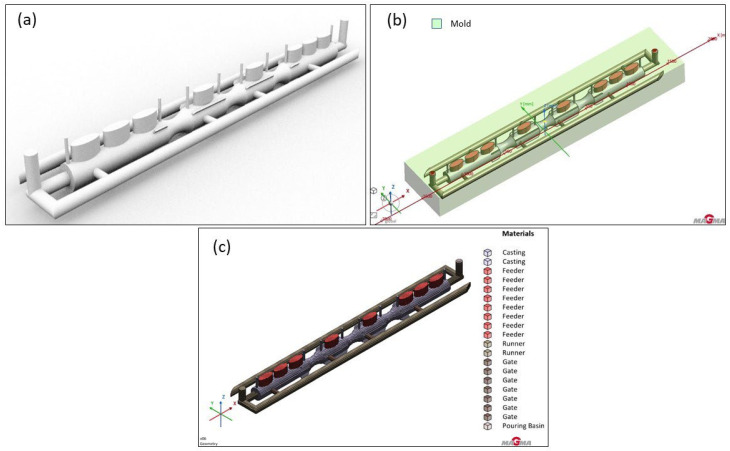
Version 4: Two runners and modified feeders: (**a**) shape of the casting with the gating system and feeders; (**b**) geometry in the simulated sand mold; (**c**) mesh with the gating system.

Previous simulations demonstrated that, especially for steels with a high tendency for shrinkage, feeders are essential. In Version 3, feeders were indeed used, but their size proved to be significantly undersized, limiting their efficiency to approximately 40%. For this reason, the next series of simulations introduced major changes in both the size and shape of the feeders, as the previous design did not sufficiently match the placement of hotspots within the casting. Instead of cylindrical feeders, oval ones were selected, better contouring the areas with hotspots. Their volume was also substantially increased (as shown in Table 2), leading to improved feeding capacity and better outcomes. These changes are expected to be particularly effective for steels where substantial feeding is required. The adjustments are visible in Figure 15.

**Figure 16:**
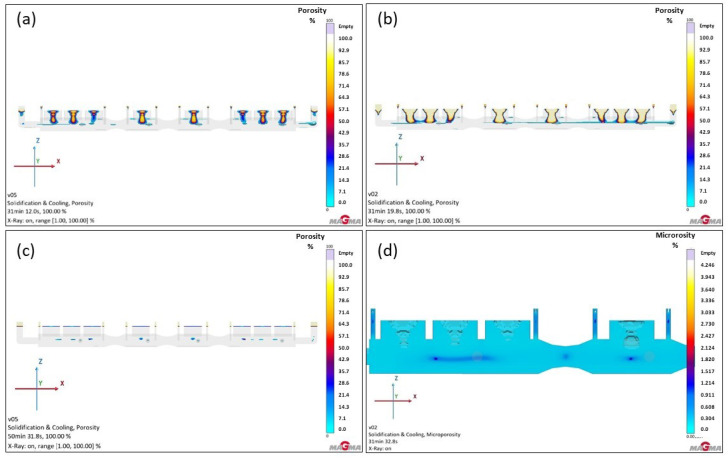
Total porosity results of version 4: (**a**) GS-34CrMo4; (**b**) GS-20Mn5; (**c**) GJS-500-7; (**d**) detail of smaller shrinkage in third feeder for GS-20Mn5.

In previous simulations, the GS-34CrMo4 material exhibited the worst results. Although it was expected that the utilization of liquid metal would be relatively low with the modified, more massive feeders, a significant improvement in casting quality was anticipated. These expectations were confirmed following the simulation. The changes proved effective, and the feeder functioned much better than in the previous Version 3, thanks to the increased volume and altered shape. As a result, porosity and shrinkage were significantly relocated to the feeder, which would subsequently be removed after production. Nevertheless, the outcome was not entirely satisfactory—approximately 25% of the defect volume remained in the casting, which was not fully optimal. The porosity percentage under the feeders in the casting reached 30–50%, indicating the need for further optimization (see Figure 16a).

Surprisingly, simulations with GS-20Mn5 material showed slightly worse results than with GS-34CrMo4. Although the defects penetrated the casting almost identically, their percentage in the casting beneath the feeders differed, in the range of around 80–90%. The microporosity visualization revealed large cavities, more extensive than in the previous version. In both cases, it was evident that defects were less prominent in the narrower parts of the furnace roll, due to the faster solidification of metal in these areas, shifting the defects.

The GJS-500-7 material demonstrated the best performance in terms of defects among the three materials, as seen in previous simulations. Nevertheless, Version 4 was simulated to assess the impact of a larger feeder volume. Simulation results of Version 4 indicated an improvement over previous simulations with this material, primarily in reducing the percentage of defects in critical casting areas, now not exceeding 20%. These critical areas are shown in light blue in Figure 16c. However, the use of such massive feeders significantly increased the liquid metal consumption, raising questions about the economic viability of Version 4 for this material, as the improvement was minimal. Given the placement and extent of defects, it is likely that even a higher defect ratio would not cause major issues during the roll’s use, as the porosity is centrally located in the roll. If further reduction in these defects is required, options could include metallurgical adjustments, changes to the pouring temperature, or the placement of external chillers, which could help reduce these types of defects further.

Compared to the previous series of simulations, where feeders were applied to the casting’s critical areas but proved undersized, this phase showed a marked improvement. The feeders were significantly enlarged, and their shape was modified to better meet the feeding requirements. For both GS-34CrMo4 and GS-20Mn5 materials, this adjustment reduced shrinkage by approximately 40%, representing a significant advancement, though it did not entirely eliminate the defect. A portion of the shrinkage still extends into the casting, complicating its practical application. Summary results of simulated Version 4 are shown in Table 6.

Completely eliminating this defect would require even larger feeders or further optimization of their shape. A possible solution could involve using a massive chiller or an elongated feeder on problematic sections of the furnace roll, which would shift the hotspot and divert shrinkage away from the roll. However, this approach would result in low utilization of liquid metal and complicate subsequent machining. For these reasons, using the GJS-500-7 material appears to be a more suitable choice, as it better meets production requirements.

### 3.5. Version 5: GJS-500-7 Simulation with Various Feeders

Based on the conducted simulations, the GJS-500-7 material—ductile iron with graphite nodules—achieved the best results. Thanks to its properties, it demonstrated less susceptibility to volumetric changes and shrinkage formation. This also reduced the need for feeders compared to steels, which have a higher tendency for shrinkage. Additionally, this material theoretically provides adequate strength and durability properties for the intended use of the final casting.

**Figure 17:**
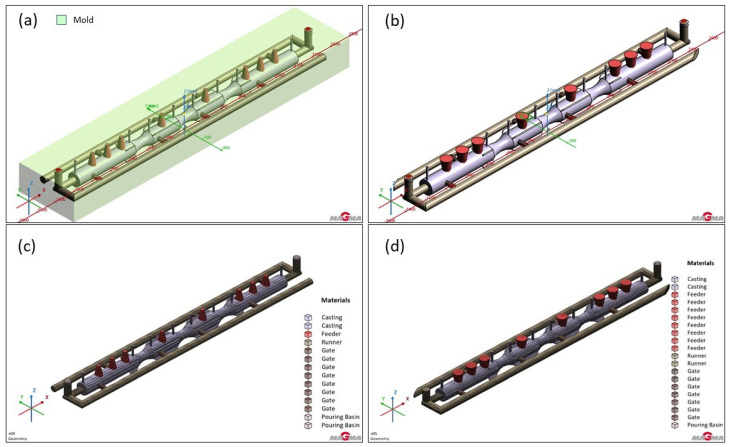
Version 5: Change in feeder shape for GJS-500-7: (**a**) conical feeder geometry in the mold; (**b**) feeder with neck geometry; (**c**) mesh for conical feeder; (**d**) mesh for feeder with neck.

**Figure 18:**
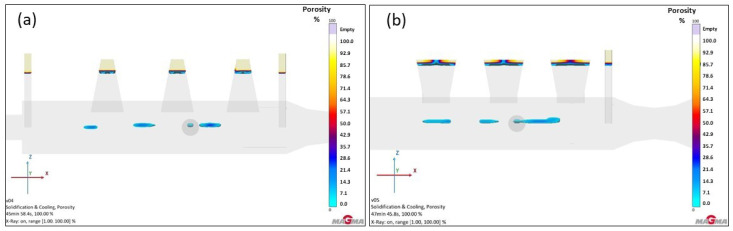
Total porosity results of version 5: (**a**) conical feeder; (**b**) feeder with neck.

In practice, minor shrinkage, primarily in the central part of the furnace roll, could be an issue. The goal, therefore, was to completely eliminate or at least minimize these defects. Initial simulations with feeders suggested that, unlike steel, where feeder volume is crucial, ductile iron benefits more from adjustments to the feeder’s shape and type. For this reason, additional simulations were performed to determine which feeder type would best suit the casting design and minimize defects. Cone-shaped feeders and necked feeders were designed, with their shapes shown in Figure 17. Parameter changes (volume and others) are detailed in Table 2. Both feeder versions and their resulting effectiveness are presented in Figure 18.

The cone-shaped feeder proved advantageous, especially for more efficient utilization of liquid metal compared to the previously assessed Version 4. Due to its conical shape, this feeder covered a larger portion of the casting’s hotspot, allowing it to supply the necessary amount of metal. When comparing porosity with the previous version, this feeder type undeniably delivered better results. Although some porosity remained in the casting, its percentage was reduced, with a maximum value of approximately 16%, which is considered low.

The results for the necked feeder were nearly identical to those of Version 4. While the color display of porosity occurrence indicated slightly higher values (a larger colored area), the percentage did not exceed 7%. In the color display, only light blue shading is visible, without the dark blue center that indicated higher porosity probability in the cone-shaped feeder. The necked feeder thus represented almost a 50% improvement over the cone-shaped feeders. However, the higher incidence of porosity suggested poorer coverage of the hotspot, which could potentially be resolved by increasing the number of these feeders. Detailed results for the feeders in Version 5 are shown in Table 7.

### 3.6. Summary of Results for All Simulated Versions

The simulations began with modifications to the furnace roll design, primarily to simplify production and prevent specific defects, such as misruns, poor metal homogeneity, or internal stresses. The proposed shape maintained uniform wall thickness throughout the casting and minimized sharp edges and transitions that could serve as stress initiators in the future. The only element retained from the original design was the thin fins, serving as guide rails to ensure the proper direction of movement for the heat-treated pressure cylinder.

After modifying the design, attention turned to the gating system and mold cavity filling. Given the challenging shape of the casting, especially its length, issues such as misruns were anticipated. This type of defect arises from rapid cooling of the metal or an inadequately dimensioned gating system that cannot deliver metal to the mold cavity quickly enough, leading to cooling before full filling. In the first simulation, one runner and five gates were used. Although the filling was successful, the fill time reached approximately 17 s, which was too long. These initial simulations were conducted without feeders, anticipating that the metal volume for the final design would be much higher. Adding a second runner with double the gates reduced the filling time by nearly half and resulted in more uniform cavity filling. Theoretically, a single runner with five gates might suffice for ductile iron, given its lower density compared to steel, along with a reduced need for feeders, as this material showed a lower tendency for volumetric changes.

**Figure 19:**
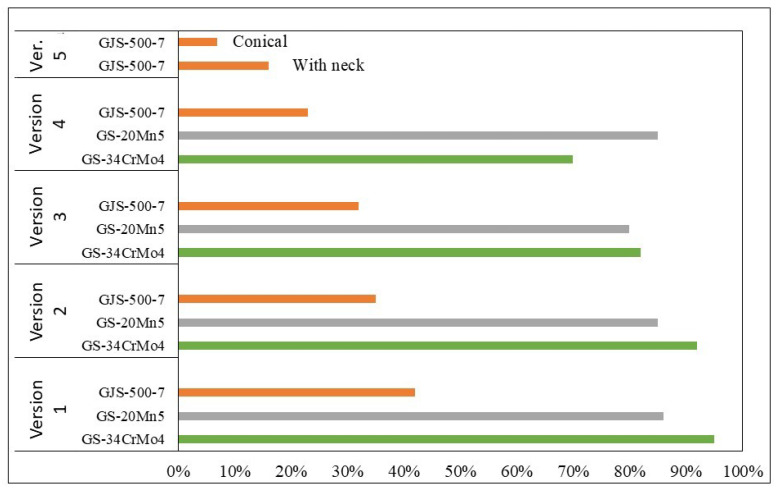
Comparison of porosity results across simulation versions and materials.

**Figure 20:**
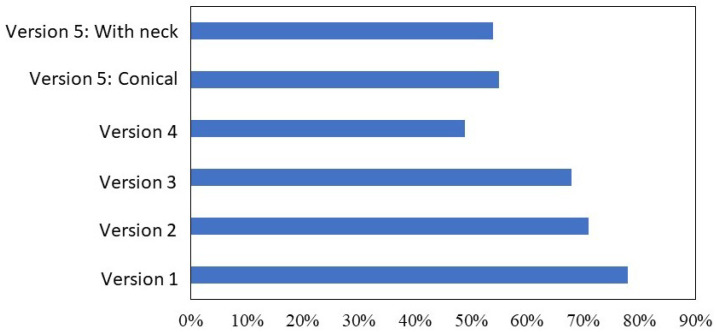
Comparison of utilization of liquid metal results across simulation versions and materials.

It was expected from the start that steel would require feeders, which was confirmed during the initial simulations. Comprehensive graphs in Figure 19 and Figure 20 showed high levels of porosity, which was expected for the early simulations. The use of initial feeders did not significantly reduce porosity, indicating the need for larger feeders. The feeder shape was subsequently changed from circular to oval, which better covered the hotspot distribution, and the increased volume provided improved feeding of liquid metal. Of the total 16 simulations, the best results were achieved with GJS-500-7 ductile iron. Although its volumetric shrinkage was the lowest, some porosity remained in the center of the furnace roll. The best outcomes were observed with the cone-shaped feeders, where porosity ranged between 7–10%, and liquid metal utilization was more efficient than with the larger feeders for steel. The lower porosity with cone-shaped feeders was attributed to the faster solidification of certain furnace roll sections. Despite the anticipated better results with larger feeders in previous versions, the effect was less favorable due to the shifting of the hotspot, which slowed metal solidification in specific furnace roll areas, particularly in sections with recesses for the cylinder.

Statistical significance of all 16 simulations is not relevant here, as the software calculates something different each time. Each version has different settings, leading to different results. Distinct parameters thus produce different outcomes, which the user then evaluates to determine the most suitable option. With identical settings, the result will be the same after each calculation.

In both steels, porosity was significantly present, and despite using very massive feeders, it was not possible to optimize the casting to be free of defects. This approach would also require high material consumption, which would be inefficient with uncertain results. The best outcome was achieved with the GS-34CrMo4 material using very massive feeders (see Figure 16a). During the simulation, it was clear that the feeder functioned correctly, as a large portion of the shrinkage moved into it. However, the result was not entirely defect-free, though it was the best among the steel simulations. This was due to both the material, which had a lower tendency for volumetric changes than GS-20Mn5, and the effectiveness of the massive feeder. Further increasing the feeder volume could theoretically yield a defect-free casting, but its production would be highly inefficient, as the amount of metal for the feeder would nearly equal the metal used in the casting itself.

According to the results obtained, the most suitable production version for manufacturing the furnace roll appeared to be the use of GJS-500-7, a ductile iron. Although its strength was the lowest among all materials, it was sufficient for the intended purpose. All defects were also concentrated in the central part of the casting near the geometric axis, where they posed a lower risk due to low mechanical stress (mainly heat stress) when used. In furnace rolls used in annealing furnaces, which are subjected to thermal cycling and surface wear, internal defects such as minor porosity and shrinkage cavities along the central axis of the roll may have minimal impact on functionality. This assumption is based on several factors. Defects located along the central axis are distant from the surface, which undergoes direct mechanical and thermal stresses; therefore, their influence on surface properties and wear resistance is limited. The primary stress on furnace rolls is thermal cycling and surface wear, so internal defects, if not extensive, usually do not negatively impact the roll’s ability to withstand these conditions. Minor porosities and shrinkage cavities often do not significantly affect the material’s mechanical properties, particularly if they are not associated with surface cracks or other defects that could initiate failure. According to the study [47], internal defects and stresses can affect the dimensional stability of castings, but the impact of these defects largely depends on their location and specific operational conditions. This study emphasizes that defects along the central axis can be of less concern if they are not subjected to direct stress. Another source [48] discusses how internal defects like porosity and shrinkage can impact the mechanical properties of castings. The study highlights that the criticality of these defects depends on their size, distribution, and the operational stresses encountered. The results of these studies suggest that minor internal defects along the central axis of a furnace roll will not have a significant impact on functionality if the roll is only subjected to thermal and surface wear. However, to ensure reliable performance, it is recommended to conduct a detailed analysis that includes the type, size, and location of defects, as well as the operational conditions under which the roll will be used.

Additional advantages included adequate thermal resistance and a lower tendency for shrinkage, which was crucial for this type of product with limited options for feeding and design adjustments. Other benefits included excellent foundry properties and potentially lower production costs due to the material’s lower density, resulting in a lighter raw casting and subsequently reduced molten metal requirements. It is also worth noting the theoretical reduction in finishing time due to a decreased need for feeder application or the use of necked feeders, which represent a smaller area on the casting that would require grinding.

Potential inaccuracies in the software may be caused by outdated or inaccurate data. Furthermore, discrepancies between practical parameters and those used in the simulation can contribute to inaccuracies. Additionally, the fineness of the computational mesh is a factor, as an overly coarse mesh can yield imprecise results. Moreover, exceptional physical and specific real-time conditions in foundry operations can affect the results differently than in the simulation. Last but not least, inaccuracies may arise under pressure for rapid computation. Simplifying parameters can speed up the acquisition of results, but the outcome may not be entirely precise. The similarities and settings across programs are discussed in articles [33,49], and minor deviations found in the programs have been corrected.

Inaccuracies are not included in the experiment because the risks described above have been eliminated. However, two areas must not be overlooked: adherence to parameters, operator supervision, and alignment of results with initial production. If all production process conditions/settings based on the Magma results are not followed in actual production, deviations may occur. By strictly adhering to all settings, simulation results can be compared with real production, allowing for fine-tuning of software settings for subsequent calculations, which will then better align with the specific production environment and thus be even more accurate. Without comparison to specific in-plant production, results may differ slightly, though not enough to undermine reliability. Inaccuracies are more likely expected from human factors, such as variations in the operation of the pouring ladle, etc. This can be minimized, for example, by consistently using the same operators.

## 4. Conclusions

The goal of the simulations and subsequent design modifications was to identify the optimal production technology for the furnace roll with minimal internal defects that could impact its functionality. The novelty of the numerical modeling approach lies in the application of an iterative simulation process to optimize the casting and gating design of an already industrially used, geometrically fixed component. Despite strict production limitations that did not allow significant geometric modifications, the simulations enabled the identification and comparison of defect tendencies (porosity and shrinkage) across different materials, and the optimization of feeders and gating systems to achieve defect minimization. This approach demonstrates a practical integration of numerical simulation into real foundry production, directly supporting decision-making in material selection and process optimization for a complex, large casting such as the furnace roll. The most significant findings are as follows:

1. It was necessary to modify the casting design to minimize sharp edges and transitions, thereby reducing susceptibility to internal stresses. However, key features, such as guide rails, essential for correctly directing the movement of the cylinder in the quenching furnace, needed to be retained.
2. Given the complex casting shape, simulations identified that the most suitable approach was to use two runners with gating channels, ensuring uniform and sufficiently rapid mold filling, which is crucial for achieving a quality casting.
3. Simulations revealed that the cast steel alloys (GS-34CrMo4 and GS-20Mn5) exhibited a high tendency for linear and volumetric shrinkage, which is typical for cast steel in general. However, despite the fact that it was possible to eliminate extensive volumetric shrinkage, the large amount of shrinkage porosity was still present and difficult to eliminate, even with various types of feeders. In contrast, GJS-500-7 ductile iron showed a much lower level of these defects due to its reduced tendency for volumetric changes.
4. In the case of steels, feeders were inefficient due to the fixed casting design, as they required large amounts of liquid metal, resulting in an uneconomical production process with potentially complex finishing. However, for GJS-500-7, a smaller feeder volume was sufficient to achieve the desired quality, which could hypothetically contribute to greater efficiency and cost-effectiveness in production.
5. Of the sixteen simulations conducted, GJS-500-7 (ductile iron) yielded the most satisfactory results. Defects were minimized to a nearly negligible level, allowing it to be used in practice without issues. All defects were also concentrated in the central part of the casting near the geometric axis, where they posed a lower risk due to low mechanical stress (mainly heat stress) when used. Due to its lower shrinkage and reduced defect occurrence, this material was better suited to the specific properties of this casting.

Based on the simulation results, GJS-500-7 appears to be the most suitable version for manufacturing the roll. Although it has lower strength than steel, its thermal resistance, castability, low shrinkage tendency, and reduced propensity for internal porosity—which is manageable through casting techniques—make it an ideal choice for this type of product. Additionally, the lower weight of ductile iron further enhances production cost-effectiveness. This solution represents the optimal choice in terms of quality, cost-effectiveness, and feasibility of production.

The results of the selected most suitable simulation variants are currently being verified in real practice with an industrial partner. The verification itself is a very time-consuming process, as the experimental validation in a foundry requires time-intensive adjustments to the casting technology, including modifications to the pattern equipment. Furthermore, the functionality of the casting and improved service life compared to the original welded part must be validated under cyclic loading in specific operations (use in an annealing furnace). At present, consent has not been granted by the operator of the castings to publish the partial results of the experimental validation, as these are still ongoing.

## Figures and Tables

**Table 1:** Properties of the simulated materials used/thermophysical properties.

Material Type	Solidus Temperature	Liquidus Temperature	Thermo Criteria Temperature	Latent Heat	Surface Tension Coefficient	Viscosity at Liquidus Temperature
	[°C]	[°C]	[°C]	[kJ/kg]	[N/m]	[m^2^s]
GS-34CrMo4	1393	1496	1498	248	1.4960	8.0356 × 10^−7^
GS-20Mn5	1428	1506	1508	256	1.4960	8.0476 × 10^−7^
GJS-500-7	1166	1169	1166.3	210	1.3	1.6000 × 10^−6^

**Table 2:** Overview of simulated versions and their parameters.

Version	Temp. Init.	Material	Description Feeders/Gates	Mass Casting	Mass Feeder	Volume Feeder Pcs	Mass Gating System	Pouring Time
[No.]	[°C]			[kg]	[kg]	[cm^3^]	[kg]	[s]
**1**	1600	GS-34CrMo4	9 (cylinder)/5	187.0	9.7	155	64.3	27.5
**1**	1630	GS-20Mn5	9 (cylinder)/5	186.3	9.7	155	64.3	27.8
**1**	1400	GJS-500-7	9 (cylinder)/5	184.1	9.5	155	63.5	28.4
**2**	1600	GS-34CrMo4	0/10	186.5	0	0	129.3	15.7
**2**	1630	GS-20Mn5	0/10	185.7	0	0	128.8	15.7
**2**	1400	GJS-500-7	0/10	177.5	0	0	128.6	15.7
**3**	1600	GS-34CrMo4	10 (cylinder)/10	186.6	10.8	155	128.9	17.7
**3**	1630	GS-20Mn5	10 (cylinder)/10	185.8	10.7	155	128.4	18.0
**4**	1400	GJS-500-7	10 (cylinder)/10	183.6	10.6	155	126.9	18.0
**4**	1600	GS-34CrMo4	8 (elipsoid)/10	175.7	54.4	984	126.8	20.3
**4**	1630	GS-20Mn5	8 (elipsoid)/10	175.7	42.0	760	126.3	20.3
**4**	1400	GJS-500-7	8 (elipsoid)/10	173.7	41.4	758	125.0	20.3
**5-conical**	1400	GJS-500-7	8 (truncated cone)/10	177.5	16.4	300	127.3	16.7
**5-with neck**	1400	GJS-500-7	8 (inverted truncated cone/with neck)/10	181.5	25.3	463	125.0	18.7

**Table 3:** Results of the version 1 simulation with a single runner.

Material	Utilization of Liquid Metal [%]	Porosity [%]
GS-34CrMo4	78	95
GS-20Mn5	78	86
GJS-500-7	78	42

**Table 4:** Results of the version 2 simulation with two runners.

Material	Utilization of Liquid Metal [%]	Porosity [%]
GS-34CrMo4	71	92
GS-20Mn5	71	85
GJS-500-7	71	35

**Table 5:** Results of the version 3 simulation with two runners and feeders.

Material	Utilization of Liquid Metal [%]	Porosity [%]
GS-34CrMo4	68	82
GS-20Mn5	68	80
GJS-500-7	68	32

**Table 6:** Results of the version 4 simulation with two runners and modified feeders.

Material	Utilization of Liquid Metal [%]	Porosity [%]
GS-34CrMo4	49	70
GS-20Mn5	49	85
GJS-500-7	49	23

**Table 7:** Results of the version 5 simulation of GJS-500-7 with various feeders.

Feeder Shape	Utilization of Liquid Metal [%]	Porosity [%]
Conical	54	7
With neck	55	12

## Data Availability

The original contributions presented in this study are included in the article. Further inquiries can be directed to the corresponding author.
